# Modulation of TGF-β signaling new approaches toward kidney disease and fibrosis therapy

**DOI:** 10.7150/ijbs.101548

**Published:** 2025-02-03

**Authors:** Quan Hong, Hyoungnae Kim, Guang-Yan Cai, Xiang-Mei Chen, John Cijiang He, Kyung Lee

**Affiliations:** 1 Department of Medicine, Division of Nephrology, Icahn School of Medicine at Mount Sinai, NY, USA.; 2 Department of Nephrology, Chinese PLA General Hospital, Chinese PLA Institute of Nephrology, State Key Laboratory of Kidney Diseases, National Clinical Research Center of Kidney Diseases, Beijing, China.; 3 James J. Peters Department of Veterans Affairs Medical Center, Bronx, NY, USA.

**Keywords:** Kidney fibrosis, CKD, DKD, TGF-β, ALK5, ALK1, TGFBR2, Smad3, HIPK2, LRG1.

## Abstract

The prevalence of chronic kidney disease (CKD) is increasing worldwide, posing a significant healthcare challenge. Despite the immense burden of CKD, optimal therapies remain limited in impact. Kidney fibrosis is a common mediator of all CKD progression, characterized by excessive extracellular matrix deposition and scarring of kidney parenchyma. Transforming growth factor-β (TGF-β) is a potent pro-fibrotic cytokine that signals through canonical and non-canonical pathways to promote kidney cell damage and fibrosis progression, thus garnering much interest as an optimal therapeutic target for CKD. However, the clinical translation of TGF-β inhibition in CKD and other disease settings has faced substantial challenges, particularly due to the highly pleiotropic effects of TGF-β in organ homeostasis and disease. Here, we review the kidney cell-specific biological effects of TGF-β signaling, discuss the current challenges in therapeutic targeting TGF-β in CKD, and provide the rationale for alternative targeting strategies of TGF-β signaling as potential approaches in CKD therapy. Selective inhibition of TGF-β signaling modulators to fine-tune TGF-β inhibition without a broad blockade may lead to new and safer treatments for CKD.

## Introduction

Chronic kidney disease (CKD) is estimated to affect more than 800 million individuals worldwide and is associated with high morbidity and mortality 1. CKD tends to progress slowly to end-stage kidney disease (ESKD), requiring kidney replacement therapy (KRT) of dialysis or kidney transplantation as the main treatment to prolong survival. As the number of patients requiring KRT is expected to rise sharply over the next few decades 2, there is an urgent need to develop effective treatments for CKD and fibrosis to halt the progression to ESKD.

Transforming growth factor-β (TGF-β) is a well-established pathogenic driver of kidney disease development 3, 4. Early human clinical studies have demonstrated the increased expression of TGF-β ligands in diseased kidneys, and numerous experimental studies have shown the causal link of TGF-β in inducing glomerulosclerosis and tubulointerstitial fibrosis. As such, its inhibition has garnered much interest as a therapeutic option for CKD, such as diabetic kidney disease (DKD) and focal segmental glomerulosclerosis (FSGS). However, the clinical translation of pharmacologic TGF-β inhibition has not been as successful in CKD as anticipated 5, 6. While the lack of success of anti-TGF-β antibodies for kidney disease therapy may be multifactorial, a key factor may involve the complex and pleiotropic cellular actions of TGF-β that are carried out in a cell type- and context-dependent manner, posing a significant clinical challenge. For instance, while hyperactive TGF-β signaling exerts potent pro-fibrotic effects across multiple organ injuries, TGF-β is also required to regulate the inflammatory immune response, such that *Tgfb1*-null mice develop a multifocal inflammatory disease and early postnatal lethality 7, 8. In the context of cancer progression, TGF-β has a biphasic function such that in the initial stages of tumorigenesis, it exerts antiproliferative and tumor-suppressive effects, but in later stages it contributes to malignant progression by promoting metastasis and chemoresistance 9, 10. In the developing vasculature system, TGF-β signaling can elicit divergent outcomes. Depending on the engagement of specific type 1 TGF-β receptors and downstream cognate Smad signaling molecules 11, TGF-β can induce endothelial proliferation or inhibit proliferation to promote quiescence. Similarly, TGF-β signaling is an essential instructive component of nephrogenesis and kidney development 12-16, but its persistent signaling is a major driver of glomerulosclerosis and tubulointerstitial fibrosis in kidney disease settings. Moreover, its diverse kidney cell-specific effects, both homeostatic and pathogenic, continue to emerge in kidney injury response and disease progression. Therefore, the challenge to specifically restrain the TGF-β's deleterious role in kidney disease remains a critically unmet need. This review aims to provide an integrative conceptual framework on kidney cell- and context-specific roles of TGF-β, discuss the remaining challenges in therapeutic targeting of the signaling pathway, and provide a perspective on alternative approaches and opportunities in TGF-β therapy for kidney disease and fibrosis.

## 1. TGF-β signaling in kidney homeostasis and disease

### 1.1 Overview of TGF-β signaling

TGF-β proteins, belonging to a superfamily of cytokines that comprises a large group of structurally related growth factors 17, consist of three mammalian isoforms, TGF-β1, TGF-β2, and TGF-β3. Although TGF-β1 is the most studied isoform in kidney disease settings, all three isoforms have been detected in developing and adult human kidneys and shown to have similar potential to produce extracellular matrix in cultured kidney cells 18-20.

**Figure 1:**
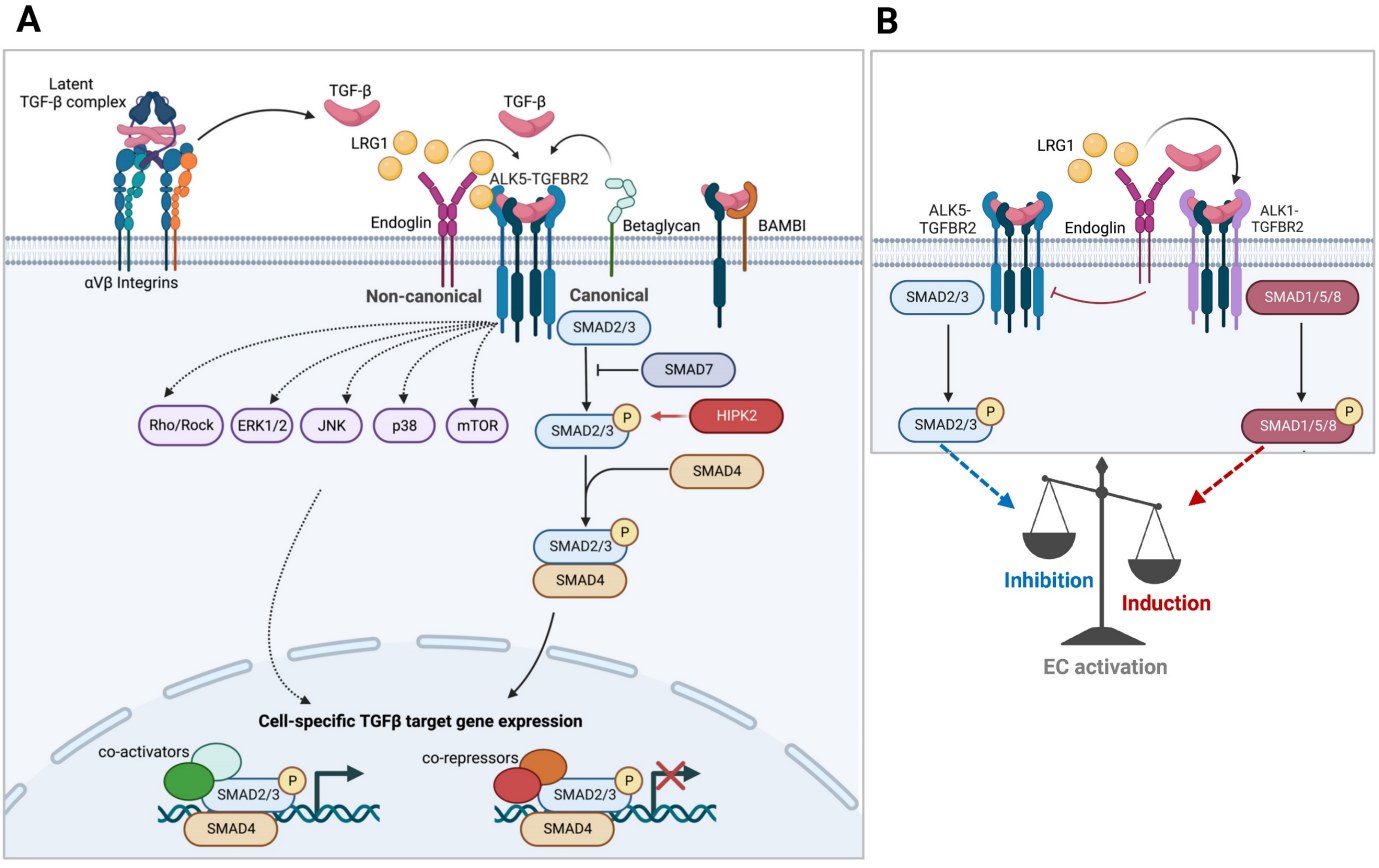
** TGF-β signaling. A.** Schematics of Smad-mediated canonical and Smad-independent noncanonical TGF-β signaling transduction are shown. Cell type and stage-specific TGF-β signaling is dictated by the expression of extracellular and intracellular signaling mediators and inhibitors and the combination of transcriptional repressors and activators present.** B.** TGF-β signaling in endothelial cells involving the interplay between two type 1 TGF-β receptors (ALK1 vs. ALK5) is shown.

TGF-β molecules are synthesized and secreted into the extracellular matrix (ECM) in an inactive form as part of a latent complex containing TGF-β, the latency-associated peptide (LAP), and latent TGF-β binding protein (LTBP) 21 (**Figure 1A**). The latent TGF-β complex remains in the ECM until triggered by signals for its release. Once activated, TGF-βs elicit cellular responses through the heterotetrameric complex of type I and type II TGF-β receptors, TGFBR1 and TGFBR2. There are two mammalian type I receptors, ubiquitously expressed TGFBR1, also known as activin receptor-like kinase 5 (ALK5), and ALK1 (also known as ACVRL1), whose expression is largely restricted to endothelial cells. Constitutively active TGFBR2 dimers, upon TGF-β binding, oligomerize with type 1 receptor dimers, leading to their phosphorylation. The activated type I receptors, in turn, relay the signal through a family of receptor-regulated Smad (R-Smad) effector proteins (Smad1, 2, 3, 5, and 8). Phosphorylated R-Smads form complexes with co-Smad (Smad4) and are shuttled into the nucleus for transcriptional regulation on numerous target genes. Because of their relatively low DNA-binding affinity, Smad complexes cooperate with an extensive repertoire of high-affinity DNA-binding transcription factors and regulators to induce or repress gene expression 22. Thus, the spatiotemporal expression and activity of transcriptional co-regulators allow the TGF-β signaling to elicit diverse biological outcomes in physiologic and pathologic contexts, albeit working through a common set of intracellular Smad proteins.

In addition to the canonical Smad-dependent signaling, activated TGF-β receptors can propagate Smad-independent, non-canonical signaling through intracellular effectors including mitogen-activated protein kinase (MAPK) family of proteins (ERK1/2, JNK, and p38 MAPK), phosphatidylinositol-3 kinase (PI3K), IκB kinase (IKK), and Src and Rho families of GTPases 23 (**Figure 1A**). The non-canonical mediators can signal independently or work in concert with Smads to generate highly context-dependent gene expression and physiologic responses. For instance, TGF-β-activated kinase 1 (TAK1), belonging to the MAP kinase kinase kinase (MAP3K) family, promotes Smad-dependent and -independent signaling in response to TGF-β. However, TAK1 signaling is not necessarily restricted to TGF-β stimuli, but it can also respond to pro-inflammatory cytokines, such as TNF-α , IL-1, and toll-like receptor (TLR) ligands, and acts as a crucial regulator of NF-κB activity in inflammatory and immune signaling pathways 24. Thus, TAK1 can propagate both TGF-β signaling and mediate the interplay of crosstalk between TGF-β and inflammatory signaling pathways in a context-specific manner.

The fine-tuning of signaling amplitude, duration, and specificity of TGF-β is also achieved by the combinatorial actions of positive and negative regulatory feedback loops, mediated by post-translational modification of signaling protein or transcriptional induction 25. For example, a set of genes rapidly induced in many cell types following TGF-β treatment encodes for inhibitory Smads (I-Smads), Smad6 and 7, as a part of a negative feedback mechanism. I-Smads inhibit the propagation of TGF-β signal transduction by interfering with R-Smad activation by competing for interaction with type 1 receptor and by recruitment of E3 ubiquitin ligases to promote degradation of type 1 receptor. TGF-β signaling also induces the expression of BMP and activin membrane-bound inhibitor (BAMBI), a pseudo-receptor with the extracellular domain related to type I receptor but lacking an intracellular kinase domain. It readily forms heterodimers with type I receptors, impeding their heteromeric complex formation with type II receptors to inhibit TGF-β signaling 26. It also synergizes with I-Smad, Smad7, to inhibit the interaction between type I receptors and R-Smads 27. Many miRNAs targeting the components of the TGF-β pathways and its downstream genes are also induced as a mechanism of feedback regulation. Conversely, in a positive feedback loop, TGF-β can further sustain and amplify its signaling by induction of genes in the signaling pathway, such as proteins involved in its activation from latency, such as integrins and matrix metalloproteinases as well as TGF-β itself, referred to as autoinduction. Thus, the feedback loops provide an additional cell- and context-dependent specificity of signaling output.

### 1.2 TGF-β signaling in kidney cell injury and disease

Increased TGF-β signaling in human CKD was observed more than 30 years ago 28-30, and numerous experimental models have well-established its pathogenic role in kidney disease and fibrosis. Examples of murine models include the transgenic mouse model with increased circulating TGF-β1 expression in the plasma (driven by albumin promoter in hepatocytes) that progressively developed glomerulosclerosis, interstitial fibrosis, and nephrotic syndrome 31 and transgenic mouse with a targeted increase of TGF-β1 in the juxtaglomerular apparatus (driven by *Ren-1c* promoter) that also developed progressive glomerular disease with reduced kidney function 32, 33. Yet, the diverse kidney cell-specific effects of TGF-β continue to emerge and underscore the strong influence of cell type and context-dependent TGF-β signaling in kidney homeostasis and disease pathogenesis. In the following sections, select studies highlighting the kidney cell-specific effects of TGF-β are discussed, mainly gleaned from genetic manipulations of TGF-β signaling components in rodent models of CKD and fibrosis.

#### 1.2.1. Epithelial cells

##### Tubular epithelial cells

Epithelial-to-mesenchymal transition (EMT) is an integral process during development and is tightly regulated in a spatiotemporal manner. Partial EMT also occurs during epithelial injury as a mechanism of wound healing. Nevertheless, its deregulation underlies organ fibrosis and cancer progression in pathologic contexts. TGF-β is among the potent inducers of EMT via Smad-dependent and independent pathways and by cooperative crosstalk with other EMT-inducing pathways, such as Notch, Wnt, Hedgehog, and TAZ/YAP 34. The actions of TGF-β on kidney tubular epithelial cells (TECs) *in vivo*, evidenced by TEC-specific overexpression of TGF-β1 or constitutively active TGFBR1, are associated with partial EMT, deregulated proliferation and cell cycle arrest, and apoptosis and tubular atrophy, all of which promote kidney fibrosis in concert with neighboring interstitial cells 35-37. Similarly, TEC-specific overexpression of leucine-rich-α-2-glycoprotein-1 (LRG1), a secreted glycoprotein that potentiates TGF-β signaling, exacerbated unilateral ureteral obstruction (UUO)- and aristolochic acid-induced kidney fibrosis in mice 38. Interestingly, in diabetic Akita mice, proximal tubule-specific *Tgfb1* overexpression not only induced tubulointerstitial fibrosis but significantly augmented albuminuria without apparent change in the GFR, which was associated with decreased megalin expression in the proximal tubules 39. This is consistent with the earlier observation that TGF-β1 can reduce megalin expression in cultured tubular cells and that TGF-β inhibition with soluble TGFBR2 (sTβRII.Fc) treatment reduces albumin excretion in streptozotocin (STZ)-induced diabetic rats 40.

Nevertheless, notwithstanding the abundance of evidence of hyperactive TGF-β signaling in driving tubulointerstitial fibrosis, emerging evidence also indicates its involvement in tubular homeostasis and appropriate response to acute kidney injury and repair. For instance, the conditional ablation of *Tgfbr2* in proximal tubules, driven by γ-glutamyl transferase (γGT)-Cre system, conferred protection against tubular apoptosis induced by mercuric chloride 41, but the same mouse model challenged to aristolochic acid-induced injury showed worsened renal damage and fibrosis development, characterized by increased proximal tubular mitochondrial injury, oxidative stress and metabolic shifting toward aerobic glycolysis, and Th17 inflammatory response 42, 43. Similarly, the loss of *Tgfbr2* or *Smad2* in collecting duct cells, driven by *Ksp*-Cre, worsened UUO-induced tubular damage and fibrosis, which paradoxically was associated with increased TGF-β availability 44, 45. However, the global loss of *Smad3* protected against UUO-induced fibrosis 46. Thus, these seemingly contrasting results highlight the potential difference in the response to TGF-β inhibition between tubular segments (proximal versus distal), between acute and chronic injury, and potentially even between TGF-β signaling components (*e.g*., Smad2 vs. Smad3). Indeed, while often acting in concert, differential or even antagonistic roles between Smad2 and Smad3 have been observed in early development gene transcription by forkhead DNA-binding protein 47, cytostatic growth inhibition in epithelial cells 48, breast cancer cell metastasis 49, growth or migration properties in pancreatic adenocarcinoma cells 50, and vertebrate neurogenesis 51. These context-specific opposing effects between Smad2 and Smad3 are thought to be driven by their differential recruitment of transcriptional co-factors and targeting of regulatory elements 52.

##### Podocytes

As a highly specialized epithelial cell type in the kidney, podocytes are an integral component of the glomerular filtration barrier, and their injury and loss are critical determinants in the development of glomerular diseases. Mechanisms of TGF-β-mediated podocyte injury, as delineated by *in vitro* studies, include epithelial-to-mesenchymal transition (EMT) and de-differentiation 53-55, cytoskeletal alterations that allow their detachment from the glomerular basement membrane 56, and apoptosis 57-59. The glomerular phenotypes of transgenic mice with increased circulating TGF-β1 included progressive glomerulosclerosis marked by reduced Wilms' tumor 1 (WT1) expression and podocyte apoptosis 31, 55, 57 . In line with these observations, the induction of podocyte-specific overexpression of constitutively active TGF-β type I receptor (*Tgfbr1*) in mice induces podocytopathy and glomerular disease development, eventually leading to kidney failure 60. The podocyte damage caused in the transgenic mice also resulted in significant mitochondrial dysfunction and oxidative stress damage in the neighboring glomerular endothelial cells (GECs), mediated by podocyte-derived endothelin 1 (EDN1). Notably, mitigating the mitochondrial injury in GECs also attenuated TGFBR1-induced podocyte loss in transgenic mice, underscoring the intricate involvement of crosstalk between signaling pathways and between glomerular cells and in kidney disease development.

In the context of diabetic kidney disease (DKD), the study by Hathaway *et al*. demonstrated the disease-promoting effects of increased TGF-β1 and the disease-preventing effects of TGF-β1 suppression 39. Using a murine model of graded expression of *Tgfb1* (10% hypomorph to 300% hypermorph) in type 1 diabetic Akita mice, the authors demonstrated that the global suppression of TGF-β1 levels prevents DKD development, while increased expression markedly exacerbates it akin to advanced human DKD 39. They also showed that the podocyte-specific TGF-β1 increase markedly worsened diabetes-induced mesangial matrix expansion, GBM thickening, podocyte foot process effacement, and kidney function impairment in Akita mice. Podocyte-specific deletion of the negative modulator of TGF-β signaling, BAMBI, similarly accentuated podocyte loss and worsened diabetic glomerulopathy in type 1 diabetic eNOS-deficient mice 61. Conversely, conditional deletion of *Smad4* in podocytes attenuated DKD in diabetic eNOS-deficient mice 62, and global *Smad3* loss also protected from podocyte injury caused by high fat diet-induced obesity in mice 63, further corroborating the importance of podocyte TGF-β signaling in promoting diabetic glomerulopathy.

#### 1.2.2. Endothelial cells

Glomerular angiogenesis, hypertrophy, and hyperfiltration are manifestations of early DKD and are associated with disease pathogenesis 64. Recent experimental evidence implicates TGF-β to be among the vascular factors promoting glomerular angiogenesis and endothelial dysfunction in early diabetic kidneys 61, 65, 66. Indeed, the global loss of type 1 TGF-β receptors (TGFBR1 or ALK1) or type 2 receptor (TGFBR2) in mice leads to early embryonic lethality due to severe vasculature development defects 67-69. *In vitro* studies have delineated that TGF-β signaling can induce either pro- or anti-angiogenic effects based on the intricate balance between the opposing actions of ALK1 and ALK5, such that ALK1-mediated activation Smad1/5 promotes endothelial proliferation and migration to promote angiogenesis, while ALK5-mediated Smad2/3 activation promotes endothelial quiescence or apoptosis in specific contexts 70-73 (**Figure 1B**). The discriminate activation of ALK1 versus ALK5 in endothelial cells by TGF-β is contextually determined in part by cell-specific expression of co-receptors and extracellular signal modulators, such as co-receptor endoglin and secreted molecule LRG1 that preferentially engage with TGF-β and ALK1 complex 74-76. Moreover, although ALK1-Smad1/5 can antagonize ALK5-Smad2/3 signaling, ALK5 activity is also required for optimal ALK1 activity in endothelial cells *in vitro* 77, suggesting a highly dynamic and integrated regulation of endothelial TGF-β signaling *in vivo*. Therefore, it is not surprising that the activation of both Smad1/5 and Smad2/3 are increased in GECs of diabetic mice 65. Endothelial-specific loss of BAMBI, a negative modulator of TGF-β signaling, resulted in aggravated diabetic glomerulopathy in STZ-induced diabetic mice, marked by GEC proliferation and increased oxidative stress 61. Similarly, a global loss of LRG1, a secreted glycoprotein whose expression is highest in GECs among endothelial cells in murine kidneys, reduced diabetes-induced GEC proliferation and oxidative stress injury to attenuate DKD 65, 66. Notably, despite the increased number of GECs in diabetic mice, gene expression analysis indicated that proliferation and apoptosis were occurring concurrently and that both processes were attenuated by reducing TGF-β signaling 66, 78, 79. Thus, it is likely that the balance of Smad1/5 and Smad2/3 pathways promotes diabetic GEC injury and contributes to DKD progression. As advanced kidney diseases are marked by capillary rarefaction, whether and how TGF-β signaling affects GEC loss in advanced DKD remains to be better elucidated.

TGF-β signaling is also implicated in endothelial-to-mesenchymal transition (EndMT) and peritubular capillary rarefaction, a common feature of progressive kidney disease and fibrosis progression. The induction of constitutively active TGFBR1 expression in endothelial cells in transgenic mice triggered cutaneous, visceral, and microvascular fibrosis, including in the kidney 80. Conversely, partial ablation of pan-endothelial *Tgfbr2* attenuated interstitial fibrosis following folic acid- and UUO-induced kidney injury in mice, which was associated with reduced EndMT and improved microvascular density and patency, and with reduced Smad2 activation but enhanced Smad1/5 activation in peritubular capillary cells 81. Similarly, the inhibition of Smad3 in streptozotocin-induced diabetic mice reduced EndMT of peritubular capillaries and interstitial matrix deposition 82. Thus, these findings support the notion that the overactive TGF-β signaling in injured kidneys promotes EndMT and peritubular capillary rarefaction mediated by Smad2/3 activation to promote inflammation and fibrosis development in CKD. Interestingly, ALK1 haploinsufficiency worsened UUO-induced tubulointerstitial fibrosis in mice 83 and was characterized by reduced Smad1/5 signaling in cells expressing myofibroblast markers (α-SMA and S100A4). However, as global ALK1 heterozygous null mice were used in the study and multiple cell types can express these markers in injured kidneys, further validations are required to elucidate the role of TGF-β/ALK1 specifically in the regulation of peritubular capillary stability in response to kidney injury *in vivo*. Similar to the context of vasculature development, temporally regulated ALK1 and ALK5 signaling are both likely required in peritubular capillaries for the appropriate response to kidney injury and fibrosis development.

#### 1.2.3. Mesangial cells and myofibroblasts

##### Mesangial cells

Derived from stromal mesenchyme, the mesangial cells exhibit overlapping features of various stromal cells, such as pericytes, fibroblasts, and vascular smooth muscle cells 84, 85. As such, in disease states, mesangial cell activation and matrix expansion orchestrate the development of glomerulosclerosis 86. TGF-β is a potent regulator of mesangial matrix synthesis and degradation, and mice with increased TGF-β expression or glomerular signaling invariably develop glomerulosclerosis with evident mesangial expansion 31, 39, 60. In the context of diabetic kidneys, *in vitro* studies showed that high glucose conditions can induce mesangial proliferation and further induce TGF-β expression 87-89, and *in vivo* administration of anti-TGF-β antibody or the interference with canonical TGF-β signaling (by overexpression of inhibitory Smad7, Smad3 loss, or reduction in TGFBR2) attenuates mesangial matrix expansion and diabetic glomerulopathy in mice 90-93. However, TGF-β can also confer cytoprotection of mesangial cells against cellular insults through TAK1 and p38 activation and autophagy induction 94. TGF-β-mediated autophagy also participated in collagen I degradation, highlighting the importance of specificity in TGF-β signaling mediators in modulating biological outcomes.

##### Myofibroblasts

Myofibroblasts are the central matrix-producing cells in the kidney for wound healing and fibrosis development. Various cellular sources are indicated in the origin of activated myofibroblasts in kidney disease, including kidney resident fibroblasts, pericytes, bone marrow-derived mesenchymal cells, macrophages, tubular epithelial cells, and endothelial cells 95, 96. Among these, several studies indicate that fibroblasts and pericytes are the major sources from which matrix-producing myofibroblasts originate 97-99. TGF-β is the principal initiator of matrix accumulation by the myofibroblasts, and its interference is strongly associated with a reduction in interstitial fibrosis in experimental models. For instance, the genetic ablation of *Tgfbr2* in fibroblasts, driven by α-SMA-Cre or PDGFR-β-Cre, attenuated UUO-induced kidney fibrosis in mice 100, 101. Similarly, *Smad2* ablation in fibroblasts by FSP1-Cre reduced interstitial fibrosis in diabetic mice, which was associated with reduced Smad3 and TGF-β levels 102. However, some experimental evidence indicates that TGF-β blockade in interstitial cells may not sufficiently reduce kidney fibrosis development 103 and that collagen accumulation is a necessary reparative response against kidney injury 104. While further clarity is required to elucidate the discrepant outcomes between experimental models, they also underscore the necessary role of myofibroblast activation as an adaptive process in wound healing response to acute kidney injury, while persistent and uncontrolled myofibroblast activation promotes deleterious fibrosis development in the setting of ongoing tubular insult and vascular rarefaction 105-107.

#### 1.2.4. Immune cells

Early studies have demonstrated a strong immunomodulatory role of TGF-β in mice lacking TGF-β1, which manifested multiorgan inflammation phenotype reminiscent of autoimmune disorder 7, 8. Many studies have since demonstrated the wide-ranging effects of TGF-β on various facets of the innate and adaptive immune system 108, 109. The effects of TGF-β's actions on immune dynamics in kidney disease are only briefly discussed here, but the readers are referred to an excellent recent review that covers this area 110.

Akin to the myofibroblast activation discussed above, the initiation of the inflammatory response is also triggered as a reparative process during the early phases of kidney injury 111, 112. However, its persistent and maladaptive inflammatory response becomes a pathogenic component driving kidney disease and fibrosis development. Thus, it is not surprising that TGF-β regulation of immune cells is associated with both pro- and anti-inflammatory responses in kidney disease and fibrosis. For instance, an aspect of TGF-β's actions on monocytes is facilitating their recruitment to the site of injury and the production of pro-inflammatory mediators 108, 109. The genetic deletion of *Tgfbr2* in macrophages (by CD11b-Cre) reduced macrophage infiltration and ensuing fibrosis development after acute kidney injury in mice 113. However, TGF-β also acts as an anti-inflammatory mediator by inhibiting the secretion of inflammatory molecules and promoting the alternatively activated M2 macrophage phenotype. In this manner, the adoptive transfer of macrophages modified *ex vivo* by IL-10/TGF-β ameliorated adriamycin-induced nephropathy in mice attenuated renal inflammation, which was associated with decreased CD4+ T cell proliferation and infiltration and increased regulatory T cells 114, 115. This is consistent with several studies demonstrating the renoprotective function of M2 macrophages in various kidney disease models 116-118. In the context of kidney fibrosis, some evidence also indicates that a specific subset of macrophages may contribute to the matrix-producing myofibroblast pool through the macrophage-to-mesenchymal transition 119-122. In addition to macrophage/monocytes, the pleiotropic effects of TGF-β are evidenced by its regulation of lymphocytes, dendritic cells, natural killer cells, mast cells, and granulocytes to influence the initiation and resolution of inflammatory responses and immune surveillance 108, 109. An active area of research in anti-TGF-β therapy is being pursued in oncology to restore the antitumor immune response in cancer 9, 10.

In the experimental models of anti-glomerular basement membrane glomerulonephritis (anti-GBM GN), conflicting results have been observed with the blockade of TGF-β signaling *in vivo*. In the study by Zhou *et al*., the administration of the soluble extracellular domain of TGFBR2 improved renal function and ameliorated crescent formation and interstitial fibrosis 123, whereas anti-TGF-β antibody administration exacerbated the disease in the study by Mesnard *et al.* 124. Moreover, overexpression of the latent form of TGF-β in mice also attenuated anti-GBM GN 125. These seemingly contradictory findings are likely due to the complex role of TGF-β in multiple cell types, including immune cells in CKD.

## 2. Targeting TGF-β signaling in kidney disease and fibrosis: past and current approaches

**Figure 2:**
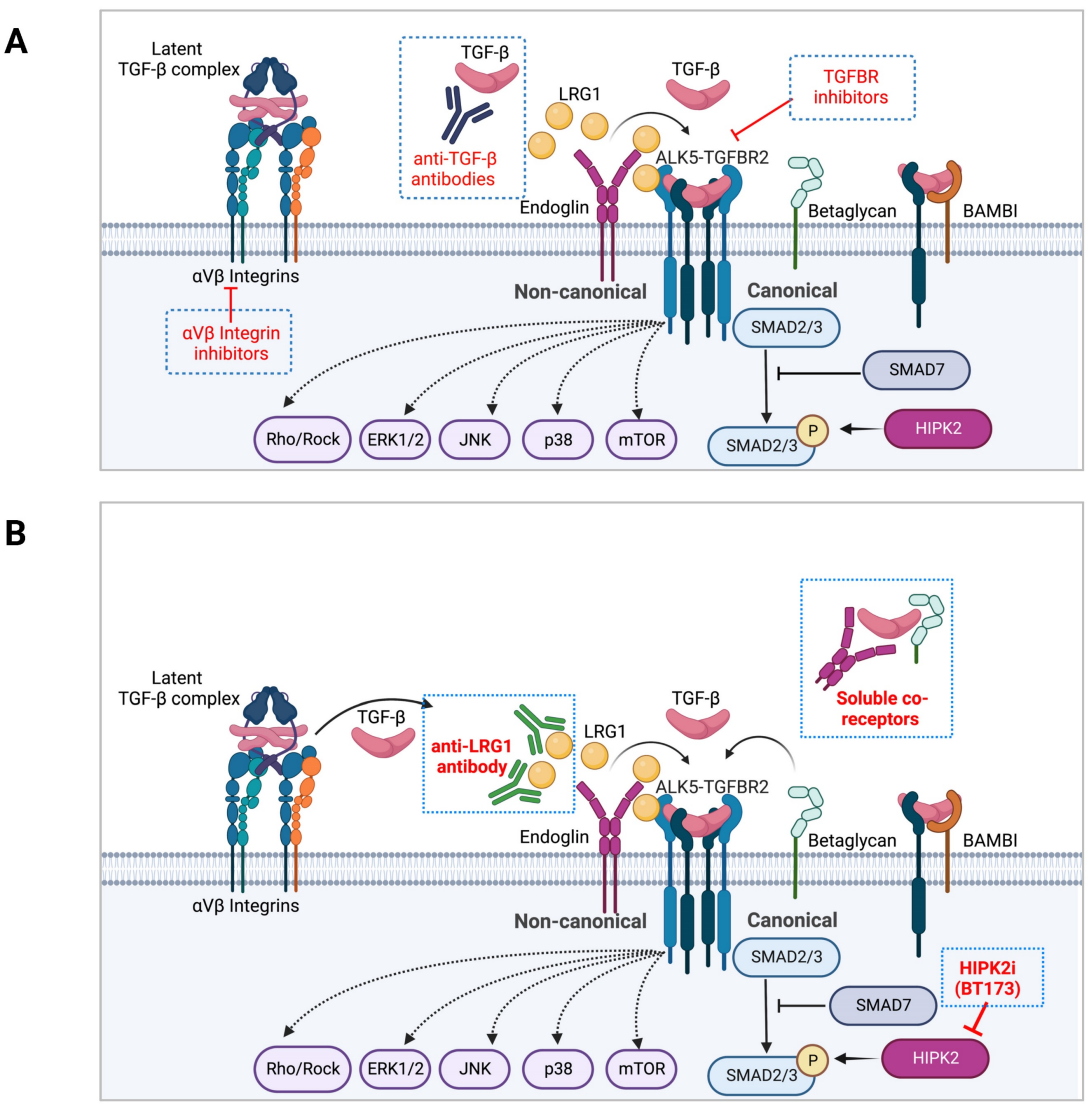
** Regulation of TGF-β signaling modulators as an alternative therapeutic approach in CKD. A.** Examples of current approaches in TGF-β signaling inhibitors are shown. **B.** Inhibitors of TGF-β signaling modulators are shown on the left as an approach to dampen the hyperactive TGF-β signaling in disease settings without a complete blockade.

As TGF-β exerts a wide range of cellular effects in various organ systems, the therapeutic modalities to dampen its effects in CKD would need to consider the balanced duality of its homeostatic and pathogenic functions of TGF-β. Drugs targeting the TGF-β pathway comprise those that inhibit its biosynthesis, activation from latency, ligand and receptor binding, receptor kinase activity, and downstream canonical and non-canonical signaling mediators. The recent advancements in anti-TGF-β therapies include function-blocking monoclonal antibodies, ligand traps, small molecule inhibitors, and antisense oligonucleotides (AONs), all currently in clinical evaluation for cancer therapy 9, 10. In this section, various approaches and modalities of TGF-β interventions in kidney disease and fibrosis development are discussed (**Figure 2A**).

### 2.1 Targeting TGF- β1 activation from latency

As TGF-β is secreted in its latent form and bound to latent TGF-β binding proteins (LTBPs), its release from its latent complex via proteolytic cleavage, mechanical forces applied by the ECM, and integrin-mediated release provides additional layers of spatiotemporal control of TGF-β signaling. In this process, α_v_-containing integrins (α_v_β_1_, α_v_β_3_, α_v_β_5_, α_v_β_6_, and α_v_β_8_) that selectively bind to the Arg-Gly-Asp (RGD) motif have emerged as a crucial regulator of TGF-β activation *in vivo* 126. Pan-α_v_ integrin small molecule inhibitors (CWHM-12/CWHM-680) were shown to attenuate renal fibrosis in mice (CWHM-12 in obstructed kidneys; CWHM-680 in aristolochic acid nephropathy) 127, 128. However, as α_v_ integrins are expressed in many cell types with wide-ranging functions, molecules targeting pan-α_v_ integrin heterodimers may be susceptible to off-target effects 129. Inhibitors of specific α_v_ heterodimers showed efficacy of TGF-β inhibition and disease progression in several experimental models. Small molecule inhibitor of α_v_β_1_ integrin, compound 8, reduced UUO- and adenine-mediated renal fibrosis development in mice 130, and α_v_β_3_ inhibitor MK-0429, originally developed for osteoporosis, attenuated albuminuria and renal fibrosis in ZSF1 diabetic rat model 131. Inhibition of α_v_β_6_ with a function-blocking monoclonal antibody attenuated renal fibrosis and inflammation in *Col4a3*-deficient Alport syndrome mice 132. Consistent with these findings, the genetic ablation of *Itgb6* protected against renal fibrosis in *Col4a3*-deficient mice and mice with obstructed kidneys 132, 133. However, *Itgb6* loss in mice also leads to exaggerated lung and skin inflammation and the development of emphysema 134, 135, highlighting the physiological importance of integrins in immune modulation. Importantly, the partial inhibition of α_v_β_6_ at a lower dose of monoclonal antibody attenuated bleomycin-induced pulmonary fibrosis in mice without exacerbating inflammation 130. No clinical outcome data yet exists for α_v_ integrin inhibitor in kidney disease, as the phase II study of humanized monoclonal anti-α_v_β_6_ antibody STX-100 (Stromedix) in chronic allograft was withdrawn (NCT0087876), and the results for phase II study of monoclonal anti- α_v_β_3_ antibody VPI-2690B (Vascular Pharmaceuticals) for in diabetic nephropathy (NCT02251067) have not been reported.

A recent study demonstrated that a monoclonal antibody, LTBP-49247, which binds selectively to TGF-β1 in latent complex with LTBPs, reduces renal fibrosis in Col4a3-null mouse model of Alport syndrome and the rat adenine model of kidney disease 136. The antibody was shown to not bind to other TGF-β isoforms, free LTBPs, or GARP- or LRCC33- bound TGF-β1 complex. As *Tgfb2* and *Tgfb3* knockout mice have cardiovascular development defects, whereas *Tgfb1*-null mice are born normally, and latent TGF-β1 are bound to GARP/LRCC33 as a latent form in immune cells, specific blockade of LTBP-bound TGF-β1 may be better tolerated.

### 2.2. Targeting TGF-β ligand

Active TGF-β signaling can further augment its signaling by autoinduction and increasing its transcript expression 137, 138, leading to a vicious cycle of signal amplification in pathogenic settings. To block the TGF-β signaling at the level of its biosynthesis, antisense oligonucleotides (AONs) targeting the translation initiation of *Tgfb1* mRNA have been used to repress ECM accumulation in rodent models of anti-Thy1 GN, diabetic nephropathy (DN), and UUO-mediated interstitial fibrosis 139-141. Pirfenidone, an anti-inflammatory and anti-fibrotic small molecule approved for the treatment of idiopathic pulmonary fibrosis (IPF), is thought to exert its effects in part by suppressing TGF-β production, although its mechanism of action is not entirely understood. Phase 2 trial is ongoing to assess the efficacy of pirfenidone in preventing CKD progression (NCT04258397).

To block the engagement of TGF-β from binding and activating its cognate receptors, ligand traps have been utilized by the introduction of soluble forms of TGF-β-interacting proteins and monoclonal antibodies against TGF-β. The fusion protein consisting of TGFBR2 ectodomain and human immunoglobulin Fc domain was utilized and shown to attenuate the development of kidney disease in several experimental models that include anti-Thy1 GN, anti-GBM GN, diabetic nephropathy, and Alport syndrome 40, 123, 132, 142. In addition, abundant pre-clinical evidence had also emerged to demonstrate the efficacy of neutralizing antibodies against TGF-β1 or all isoforms - their use attenuated kidney injury or fibrosis development in models of glomerulonephritis 143, 144, DKD in type 1 and type 2 diabetes 90, 145, 146, glomerular injury in salt-sensitive hypertension 147, puromycin aminonucleoside nephropathy (PAN)^148^, UUO- or cyclosporin A-induced tubular injury and fibrosis 149-152, and chronic allograft rejection 153. However, the efficacy of anti-TGF-β antibody Fresolimumab (1D11), a monoclonal antibody that recognizes all three TGF-β isoforms, was significantly diminished in its renoprotection if initiated during the advanced stages of DN in streptozotocin-injected rats 146. In addition, in PAN rodent model, while a lower dose of 1D11 (0.5mg/kg) was effective in attenuating glomerulosclerosis and tubulointerstitial fibrosis, this effect was largely abrogated with a higher dose (5.0mg/kg) 148, suggesting a narrow therapeutic window. In clinical settings, although Fresolimumab(1D11) was well tolerated for the treatment of focal and segmental glomerulosclerosis (FSGS) 154, it failed to achieve the pre-specified efficacy endpoints for proteinuria reduction in a randomized, double-blind phase II clinical trial (NCT01665391) 5. Similarly, the monoclonal antibody against TGF-β1, LY2382770, failed to show significant improvement in renal function and proteinuria in DN patients in the randomized double-blind phase II clinical trial (NCT11133801)^6^. The lack of success of TGF-β1 antibodies in these studies is likely multi-factorial, as even the experimental models mentioned above have demonstrated varying results based on the timing of the initiation and dosage required for optimal therapeutic intervention 146, 148.

### 2.3 Targeting TGF-β receptors

Experimental approaches to TGF-β receptor inhibition in kidney disease include small-molecule kinase inhibitors of ALK5 (TGFBR1) and function-blocking monoclonal antibodies against TGFBR2. Various ATP-competitive kinase inhibitors of ALK5 have shown efficacy in reducing renal fibrosis in rodent models. IN-1130 (IC_50_ of 5.3nM in inhibiting ALK5-mediated Smad3 phosphorylation) impeded the UUO-induced renal fibrosis development in rats 154, and R-268712 (IC_50_ of 2.5nM) attenuated glomerulosclerosis in anti-Thy1 nephritis and UUO-induced fibrosis in rats 155. SB-431542, AZ12601011, and GW788388 are select kinase inhibitors of ALK4, ALK5, and ALK7 (IC_50_ is 94nM for SB-431542 and 18nM for AZ12601011 and GW788388 in ALK5 inhibition) 156-159. Use of SB-431542 was shown to mitigate tubulointerstitial fibrosis in mouse UUO kidneys 150; AZ12601011 in fibrosis development following UUO and ischemic reperfusion injury in rats 158; and GW788388 in diabetes-induced renal fibrosis in *db/db* mice 160. Vactosertib (TEW-7197, MedPacto) is a select kinase inhibitor of ALK2, ALK4, and ALK5 (IC_50_ of 11nM for ALK5), which demonstrated a favorable safety profile and antitumor efficacy in phase I trial for advanced refractory solid tumors 161, 162 (NCT02160106). Vactosertib reduced the UUO-induced tubulointerstitial fibrosis development in mice and attenuated diabetic glomerulopathy in *db/db* mice when treated for 10 weeks 163, 164. Similarly, monoclonal antibodies against TGFBR2 as a broad TGFβ inhibition are actively pursued in various areas of cancer therapy. In the context of kidney fibrosis, a monoclonal antibody developed against TGFBR2 reduced mesangial and interstitial matrix accumulation in the model of anti-Thy1 nephritis 165. There are currently no active clinical studies of anti-TGFBR2 monoclonal antibodies for kidney disease.

However, targeting the TGF-β ligands or receptors that result in pan-TGF-β inhibition requires considerable caution in the long-term treatment of chronic diseases, such as CKD, as circumventing the potential side effects remains a significant hurdle to overcome. For instance, the anti-TGFBR2 monoclonal antibody (LY3022859, Eli Lilly) therapy for advanced solid tumors in the phase I trial was hampered in assessing the maximum tolerated dose for efficacy beyond 25mg dose level due to the uncontrolled cytokine release, despite the prophylactic therapy with corticosteroid and antihistamine (NCT01646203) 166. Treatment with the pan-TGF-β antibody, fresolimumab, was associated with the development of cutaneous carcinomas in phase I study of patients with melanoma or renal cell carcinoma 167, 168. In preclinical studies, long-term exposure to pan-TGF-β antibodies and small molecule inhibitors of ALK5, such as AZ12601011, demonstrated cardiovascular toxicity with heart valve thickening, inflammation, hemorrhage, and stromal hyperplasia 157, 169, 170. These findings are consistent with the phenotype of mice with postnatal *Tgfbr2* deletion in smooth muscle cells that displayed pronounced aortopathy 171, 172, indicating that the chronic and broad pharmacologic blockade of TGF-β can inadvertently lead to cardiovascular disease. Thus, balancing the therapeutic window and systemic off-target effects remains a significant challenge in pan-TGF-β inhibition for CKD.

### 2.4. Targeting the downstream TGF-β signaling mediators

Compared to inhibitors of TGF-β ligand and receptors, there are far fewer inhibitors of TGF-β signaling mediators in clinical testing or development. However, several experimental models have demonstrated the efficacy of Smad3 inhibition in attenuating kidney disease and ensuing fibrosis in various mouse models, consistent with findings of genetic ablation of Smad3 in different kidney cells, as discussed above. SIS3 is a purported Smad3-specific inhibitor, which reduced TGF-β-induced phosphorylation of Smad3 but not Smad2 in cultured fibroblasts 173. However, a recent study using Smad2- and Smad3-null cell lines indicates that SIS3 may be a broader inhibitor of Smad2/3 complex 174. In kidney injury *in vivo*, SIS3 delayed the development of diabetic nephropathy in streptozotocin-induced type 1 diabetic mice and type 2 diabetic db/db mice, and the inhibition of Smad3 in SIS3-treated db/db mouse kidneys was associated with increased Smad7 expression and suppression of NF-κB-mediated inflammation 82, 175. SIS3 also attenuated high fat-induced proteinuria and podocyte injury 63 and suppressed UUO-induced tubulointerstitial fibrosis development 176, demonstrating the therapeutic efficacy of SIS3 in reducing kidney injury and fibrosis in experimental models. As systemic Smad3 loss is associated with impaired mucosal immunity, chronic infection, and metastatic colorectal cancer in mice 177-179, whether the long-term use of SIS3 would be associated with any undesired effects remains to be determined.

In addition to the canonical Smad-mediated pathways, TGF-β receptors also activate other non-canonical signaling pathways such as PI3-AKT and ERK/MAPK, albeit at lower levels than by receptor tyrosine kinases (RTKs), as well as stress-activated MAPKs, p38 MAPK and JNK 23. Selective inhibitors of p38 or JNK (NPC31145, NPC31169, FR167653, and SB203580 for p38; SP600125, CC401 and CC930 for JNK) have been shown to reduce podocytes injury, inflammation and fibrosis in animal models of anti-glomerular basement membrane (GBM) glomerulonephritis, ureteral obstruction, and diabetic nephropathy 180-187. However, p38 MAPK inhibitors are adversely associated with hepatotoxicity, rash, dizziness and inhibition of erythropoietin production to affect erythropoiesis 188, 189. Studies have shown that p38 MAPK deletion mice have apparent renal structural abnormalities, including proximal tubule dilation, vacuolar degeneration, focal interstitial fibrosis, and inflammation 190, whereas JNK1 and JNK2 double knockout leads to early embryonic lethality due to neural tube defects in mice 191.

Apoptosis signal-regulating kinase 1 (ASK1) is an upstream signaling kinase of p38 MAPK and JNK in kidney diseases 192. Previous studies in animal models of kidney diseases have shown that ASK1 promotes p38 MAPK activation induced by oxidative stress, whereas ASK1 deficiency or ASK1-selective inhibition reduces p38 MAPK and JNK activation and improves kidney injury in mouse models of CKD and fibrosis 193-195. The phase 2 clinical trial to evaluate the safety and efficacy of selonsertib, an ASK1 selective inhibitor, in patients with moderate to advanced diabetic nephropathy showed no dose-dependent adverse effects by 48 weeks, but the results did not meet the primary endpoint of change in eGFR 196. A *post hoc* analysis nevertheless indicated that selonsertib slowed the kidney function decline after adjusting the confounding eGFR differences. A subsequent clinical trial evaluating the safety and efficacy of selonsertib in moderate to advanced DKD (MOSAIC, NCT04026165) demonstrated a slower eGFR decline compared to placebo but also raised potential safety concerns for acute kidney injury 197.

In addition to these cellular effectors of canonical and non-canonical signaling, various intracellular signaling mediators intersect with the TGF-β signaling pathway, thus serving as drug targets to intercept the TGF-β signaling. One such example is glycogen synthase kinase-3β (GSK-3β) 23, and recent evidence highlights a permissive effect of GSK-3β on the profibrogenic plasticity of renal tubular epithelial cells and its contribution to renal fibrogenesis in progressive CKD. Although initially identified as a regulator of glucose metabolism, GSK-3β is now understood to be a multi-functional kinase that impacts numerous biological processes. In cultured rodent tubular epithelial cells and fibroblasts, TGF-β1 can increase GSK-3β expression, and its increased expression and activity have been observed in fibrotic kidneys 198, 199. Pharmacological inhibition of GSK3 abolished TGF-β1-induced Smad3 activation *in vitro* and reduced kidney fibrosis in animal models of ischemia-reperfusion and folic acid-induced injuries 198, 199. However, currently existing inhibitors of GSK-3 have poor GSK-3β selectivity and suboptimal potency and are associated with chronic toxicities 200. Notably, several *in vivo* studies, as described above, indicated that micro-dosed lithium (one-third to one-half of neurobiological dose) bypasses the potential neurological or nephrotoxicity associated with higher doses of lithium but is still effective at conferring renoprotection against AKI and glomerular diseases in mice 199, 201-203. These results strongly indicate that lithium may be safely repurposed as a pragmatic and affordable treatment for diverse types of CKD 204.

## 3. Targeting the modulators of TGF-β signaling as alternative approaches in CKD

Although working through a common set of receptors and a limited number of intracellular effector proteins, TGF-β signaling can achieve wide-ranging cellular and biological effects in organismal development, tissue homeostasis, and disease pathogenesis. Such multifaceted repertoire of signaling capacity and its potency relies on the cell type and context-specific expression of modifiers and regulators at various steps of the signaling cascade, such as 1) regulators involved in the activation of latent TGF-β complex, 2) cell surface regulators of TGF-β and receptor complex formation, 3) regulators of Smad activation and nuclear translocation, 4) transcription factors/co-regulators that interact with Smad protein to dictate context-specific gene expression, and 5) mediators that integrate crosstalk with other cellular signaling pathways. Furthermore, TGF-β-induced genes can provide a feedback loop to either amplify or limit the magnitude and duration of the signaling output. Importantly, select modifiers can significantly amplify or limit the magnitude and duration of TGF-β signaling output in disease settings, but their loss is not detrimental at tissue or organismal levels, in contrast to the loss of TGF-β ligands or receptors that lead to severe developmental or postnatal defects. Therefore, targeting such regulators may dampen the hyperactive TGF-β signaling in disease settings to thwart the disease progression (**Figure 2B**).

### 3.1. Modulators of ligand-receptor interaction

An early point of TGF-β signaling regulation occurs by the cell surface molecules that exert context-dependent augmentation or inhibition of the active TGF-β ligand binding with its cognate receptors, such as auxiliary TGF-β co-receptors (*e.g*., betaglycan, endoglin, and BAMBI), small leucine-rich proteoglycans (SLRPs) that can bind and sequester TGF-β ligands (*e.g.*, biglycan and decorin), and secreted molecules that participate in TGF-β and receptor engagement (*e.g*., connective tissue growth factor and LRG1). A considerable influence of the above cell surface signaling modifiers on the TGF-β signaling in disease pathogenesis has been demonstrated in various models of kidney injury and disease. As SLRPs and pseudo-receptor BAMBI restrain TGF-β signaling by binding to and saturating out active TGF-β ligands, their loss markedly worsens renal fibrosis and diabetic nephropathy progression in mice 61, 205-208. Conversely, the increased soluble form of recombinant decorin attenuated the glomerular matrix accumulation in the rat model of anti-Thy1 nephritis 209, 210, and soluble recombinant betaglycan also limited the progression of diabetic nephropathy in *db/db* mice 211. However, the genetic ablation of co-receptors, endoglin and betaglycan, are also associated with detrimental defects in mice 212-215, and targeting the soluble co-receptors or SLRPs may face challenges in fine-tuning the hyperactive TGF-β signaling without a complete blockade, similarly as with soluble TGFR2 or neutralizing TGF-β antibodies that are associated with adverse toxicities.

The expression of secreted factors that regulate TGF-β signaling, such as connective tissue growth factor (CTGF) and LRG1, are often elevated in pathological conditions such as atherosclerosis, inflammatory conditions, fibrosis, and various forms of cancer 216, 217. CTGF itself is a gene induced by TGF-β signaling, and it exerts a broad range of biological influences beyond its involvement in TGF-β ligand recruitment 220. LRG1, as mentioned above, interacts with both type 1 and 2 receptors and co-receptor endoglin to facilitate their complex formation to enhance downstream signaling 76. Notably, elevated levels of circulating CTGF and LRG1 levels are both associated with worse renal outcomes in DKD patients 65, 218, 219, and are causal in kidney disease pathogenesis. Thus, podocyte-specific CTGF overexpression worsened diabetic nephropathy 222, whereas reducing CTGF expression by specific antisense oligonucleotides (ASO) improved kidney function and reduced the mesangial matrix expansion in mouse models of type 1 and 2 diabetes 221. However, CTGF inhibition may not be limited to TGF-β signaling attenuation, as it also exerts a broad range of biological influences, and its global knockout results in early perinatal lethality in mice with defective organ development and respiratory failure 212, 223. In contrast, the global LRG1 expression does not affect embryonic development or survival in adult mice, but is effective in curtailing the progression of DKD 65, 66 and tubulointerstitial fibrosis *in vivo* 38. Recently, LRG1-neutralizing antibody, Magacizumab, was shown to curtail TGF-β-mediated retinal vascular leakage and for anti-tumor activity in mice 224-226. Thus, it remains to be determined whether LRG1 blockade would be of greater utility than pan-TGF-β inhibition, particularly in kidney disease settings.

### 3.2 HIPK2, a modulator of TGF-β/Smad3 signaling potency

Downstream of the receptor activation, a substantial influence of TGF-β/Smad3 signaling is exerted by homeodomain interacting protein 2 (HIPK2) 227, 228. In addition to potentiation of TGF-β signaling, HIPK2 acts as a multifunctional regulator of diverse signaling pathways that include Wnt/β-catenin, Notch, p53, and Hippo signaling 227, 229-233. While HIPK2 protein expression is low at basal levels in the adult kidney, its expression is significantly increased in CKD of various etiologies 228. Genetic deletion of HIPK2 attenuated renal fibrosis development in various mouse models of renal fibrosis, which was associated with attenuation of TGF-β/Smad3-, Wnt/β-catenin-, and Notch-targeted genes 228, 234, and conversely, its overexpression results in exacerbated kidney injuries in experimental models of CKD and tubulointerstitial fibrosis 234-237. Notably, Liu *et al.* reported the development of a small molecule inhibitor of HIPK2, BT173, that allosterically blocks its interaction with Smad3 without altering its kinase activity 238. BT173 effectively reduced UUO- and HIV-induced renal fibrosis *in vivo*, which was associated with reduced Smad3 phosphorylation and repression of TGF-β-responsive matrix gene expression 238. More recently, BT173 analogs have been further developed for optimal physicochemical properties for clinical testing, which reduced renal fibrosis in mouse CKD models of HIVAN and Alport syndrome 239, 240. Importantly, similar to LRG1 knockout, global loss of HIPK2 does not result in developmental or immune deregulation phenotypes observed in knockout mice lacking major TGF-β signaling components. Thus, targeting HIPK2-Smad3 interaction with allosteric HIPK2 inhibitors, such as BT173, may effectively mitigate kidney fibrosis without a systemic TGF-β blockade as an alternative therapeutic strategy.

## Conclusions and prospects

Although much progress has been made in understanding TGF-β signaling in various disease contexts in the past two decades, the cell type- and disease context-specific determinants of TGF-β signaling that help define its diversity, versatility, and complexity in biological output remain to be better understood. Thus, despite the obvious potential of therapeutic targeting of TGF-β in organ fibrosis, the clinical approach to optimize the therapeutic window while minimizing the adverse effects remains a significant challenge. Nevertheless, these challenges should not preclude the remaining potential of its targeting as a fibrosis therapy but underscore a need for alternative approaches for improved specificity. In CKD settings, specific antagonisms of TGF-β signaling modulators have demonstrated effectiveness as therapeutic targets, such as LRG1 and HIPK2. As these modulators function to tip the balance toward excessive pathological signaling, their blockade is an attractive option to attenuate CKD progression and fibrosis without a complete blockade of TGF-β signaling. Therefore, future therapies aimed toward an effective combination of strategies to block the modulators of TGF-β signaling potency and duration may lead to the more successful generation of new CKD and renal fibrosis treatments.
